# Cognitive Behavioral Therapy for Treatment-Related Fatigue in Chronic Myeloid Leukemia Patients on Tyrosine Kinase Inhibitors: A Mixed-Method Study

**DOI:** 10.1007/s10880-019-09607-5

**Published:** 2019-02-12

**Authors:** Hanneke Poort, Patrick Onghena, Harriët J. G. Abrahams, Heather S. L. Jim, Paul B. Jacobsen, Nicole M. A. Blijlevens, Hans Knoop

**Affiliations:** 1 Department of Psychosocial Oncology and Palliative Care, Dana-Farber Cancer Institute, 450 Brookline Avenue, Boston, MA 02215 USA; 2 Faculty of Psychology and Educational Sciences, KU Leuven-University of Leuven, Tiensestraat 102, 3000 Leuven, Belgium; 3 Department of Medical Psychology, Amsterdam Public Health Research Institute, Academic Medical Center, University of Amsterdam, Meibergdreef 9, 1105 AZ Amsterdam, Netherlands; 4 Expert Center for Chronic Fatigue, Department of Medical Psychology, Amsterdam Public Health Research Institute, VU University Medical Center, De Boelelaan 1117, 1081 HV Amsterdam, Netherlands; 5 Department of Health Outcomes and Behavior, Moffitt Cancer Center, 12902 Magnolia Drive MRC-SCM, Tampa, FL 33612 USA; 6 Division of Cancer Control and Population Sciences, National Cancer Institute, 9609 Medical Center Drive, Bethesda, MD 20892 USA; 7 Department of Hematology, Radboud University Medical Center, Geert Grooteplein Zuid 8, 6525 GA Nijmegen, The Netherlands

**Keywords:** Fatigue, Chronic myeloid leukemia, Cognitive behavioral therapy, Single-case experiment, Mixed methods

## Abstract

Treatment-related fatigue significantly limits quality of life among chronic myeloid leukemia (CML) patients receiving tyrosine kinase inhibitors (TKIs), yet no interventions to reduce this symptom have been studied. We examined preliminary feasibility and efficacy of cognitive behavioral therapy for TKI treatment-related fatigue in patients with CML. We used a mixed methods convergent design and collected quantitative data through randomized single-case experiments. We included CML patients receiving TKIs and reporting severe fatigue. Within each participant, we compared CBT to a no-treatment baseline period. Fatigue severity was measured weekly with the Checklist Individual Strength. Fatigue scores were subjected to visual analyses and randomization tests for single-case experimental designs. We conducted qualitative interviews after study participation and focused on feasibility and efficacy of CBT. Visual inspection of line graphs indicated downward trends in the expected direction for fatigue in two of the four participants. The test statistics showed a decrease in fatigue severity for all participants but randomization tests did not reach statistical significance (overall *p* = 0.18). Participants reported qualitative improvements in level of functioning and coping with fatigue. CBT was considered feasible and acceptable for severely fatigued CML patients. Our study provided preliminary evidence for the feasibility and acceptability of CBT for severely fatigued CML patients receiving targeted therapy. We recommend further efficacy testing of this promising intervention in a pilot randomized controlled trial.

## Introduction

Chronic myeloid leukemia (CML) is a type of cancer that starts in the blood-forming cells of the bone marrow. While CML cannot be cured, with modern treatments, such as targeted therapies, it is often possible to control CML for many years (Bower et al., 2016). Targeted therapies are a new generation of cancer drugs designed to interfere with molecular targets critical for cancer growth and progression. Unlike chemotherapy, targeted therapy is a more specific treatment that alters functions of cancer cells and does not affect healthy cells. Therefore, side effects of targeted therapies are different from standard chemotherapy. Tyrosine kinase inhibitors (TKIs) are an example of a successful targeted therapy and the most common treatment for CML. Since the introduction of Imatinib in 2001 as the first TKI approved for CML (Druker et al., 2001), several other TKIs have been developed to treat CML. The management of CML with TKIs has resulted in a life expectancy for these patients similar to the general population (Bower et al., 2016). TKI therapy is generally given daily for many years and may even continue for one’s entire life. While recent studies have suggested that some patients with prolonged deep molecular responses may be able to safely discontinue TKIs (Hochhaus et al., 2017; Mahon et al., 2010; Rea et al., 2017), this approach is currently not recommended outside the context of a clinical trial (Short & Jabbour, 2017). TKIs are generally much better tolerated than previous treatments, yet patients do experience significant side effects.

Cancer-related fatigue is a common side effect among all cancer patients and defined as “a distressing, persistent, subjective sense of physical, emotional, and/or cognitive tiredness or exhaustion related to cancer or cancer treatment that is not proportional to recent activity and interferes with usual functioning **(**Bower et al., 2014).” Fatigue is one of the five most severe side effects in CML patients treated with TKIs and the major factor limiting quality of life (Efficace et al., 2013; Williams et al., 2013). In addition, fatigue is associated with reduced adherence to TKIs (Eliasson, Clifford, Barber, & Marin, 2011; Unnikrishnan et al., 2016). This fatigue must be managed not only to improve quality of life, but also to ensure adherence to the medication, which is critical for TKI effectiveness. It is therefore surprising that there are no published intervention studies addressing targeted therapy-related fatigue. Fortunately, several non-pharmacological interventions for fatigue have been studied among patients treated with standard therapies (e.g. chemotherapy, radiotherapy) for cancer. Among these, cognitive behavioral therapy (CBT) has been specifically cited as an example of an empirically supported strategy (Bower et al., 2014). Our research group demonstrated clinically significant improvements in fatigue and functional impairment after CBT in disease-free cancer patients treated with surgery, chemotherapy, and/or radiotherapy who reported severe fatigue prior to enrollment in the study (Abrahams et al., 2017; Gielissen, Verhagen, Witjes, & Bleijenberg, 2006). Positive treatment effects of CBT sustained at long-term follow-up (Gielissen, Verhagen, & Bleijenberg, 2007). However, these and other intervention studies for post-cancer fatigue did not include patients receiving targeted therapy and, thus, whether CBT is also effective in reducing fatigue in patient populations receiving more chronic cancer treatment with TKIs remains unclear.

In a previous study, we adapted our post-cancer CBT intervention for use in CML patients experiencing targeted therapy-related fatigue (Poort et al., 2018). The original intervention is based on the cognitive behavioral model of cancer-related fatigue (Gielissen et al., 2006; Servaes, Gielissen, Verhagen, & Bleijenberg, 2007). According to this model, cancer and its treatment are initial triggers for fatigue, yet after completion of cancer treatment, fatigue-related behaviors and beliefs can perpetuate fatigue. We assumed that this model also applied to CML patients with targeted therapy-related fatigue with the important difference that these patients have an ongoing trigger for fatigue (i.e. chronic TKI treatment). The CBT intervention consists of several modules that focus on the identified perpetuating factors of cancer-related fatigue (e.g. sleep dysregulation, fatigue catastrophizing, fear of disease recurrence) and is tailored to individual patients’ needs. CML patients, healthcare providers, and CBT experts participated in a systematic and stepwise adaptation process. We found that patients needed more information about their disease and treatment and, therefore, developed a new psycho-educational module. In addition, we added specific topics that are relevant to this patient population, such as adherence to chronic TKI treatment and coping with fear of increased disease activity (Poort et al., 2018).

The next step involved preliminary efficacy testing of the adapted intervention. Therefore, the aims of this mixed methods study were to explore feasibility and efficacy of CBT for targeted therapy-related fatigue using replicated single-case experiments (SCEs) and interviews.

## Methods

### Participants

We recruited patients diagnosed with CML and receiving treatment with a TKI for at least 3 months. Hematologists from the Department of Hematology at the Radboud University Medical Center in the Netherlands referred potential participants between March 2015 and May 2016. Eligible patients were at least 18 years old, able to speak and read Dutch, and reported severe fatigue (Checklist Individual Strength [CIS], score subscale fatigue severity ≥ 35). Patients receiving current treatment for a psychiatric disorder and those with a somatic or treatable cause for severe fatigue (other than TKI treatment) were excluded from participation in the study. The Research Ethics Committee of the Radboud University Medical Center approved the study (CMO Arnhem-Nijmegen, No. 2014-1403). We obtained written informed consent from all participants.

### Design

We used a mixed-method convergent design to collect quantitative and qualitative data on the efficacy of CBT for targeted therapy-related fatigue in parallel (Fetters, Curry, & Creswell, 2013). Quantitative data were collected through SCEs. The goal of a SCE is to determine whether a causal relationship exists between an independent variable (e.g. no CBT vs. CBT) and a dependent variable (e.g. fatigue). The dependent variable is generally measured repeatedly across and within all experimental time periods, known as *phases* (e.g. baseline vs. treatment). Experimental control is demonstrated if the changes in the dependent variable follow the introduction of the treatment (Smith, 2012). Adding a randomization component improves the internal validity of the findings (Kratochwill & Levin, 2010). SCEs provide a rigorous and methodologically sound alternative to group designs (Barlow, Nock, & Hersen, 2008; Kazdin, 2010; Kratochwill & Levin, 2010). This approach is particularly appropriate when unaddressed areas are explored and pilot data are generated or when studying small-*n* populations such as CML patients (Rohrbacher & Hasford, 2009). In contrast to group designs, SCEs do not require a significant amount of resources or participants, thus providing a cost-effective approach to explore whether CBT is potentially efficacious in reducing targeted therapy-related fatigue.

In this study, replicated AB single-case experimental designs with intervention start-point randomization were implemented. SCEs provide the strongest evidence possible about the efficacy of an intervention in an individual patient (Kratochwill et al., 2010). Combining the results of replicated experiments allows ascertaining an intervention effect for a patient population. We aimed to have at least five completed SCEs. Although there is no formal agreement about how many replicated experiments are needed, a conceptual norm of at least three demonstrations of an intervention effect across participants has been recommended (Horner et al., 2005; Kratochwill & Levin, 2010).

**Fig. 1:**
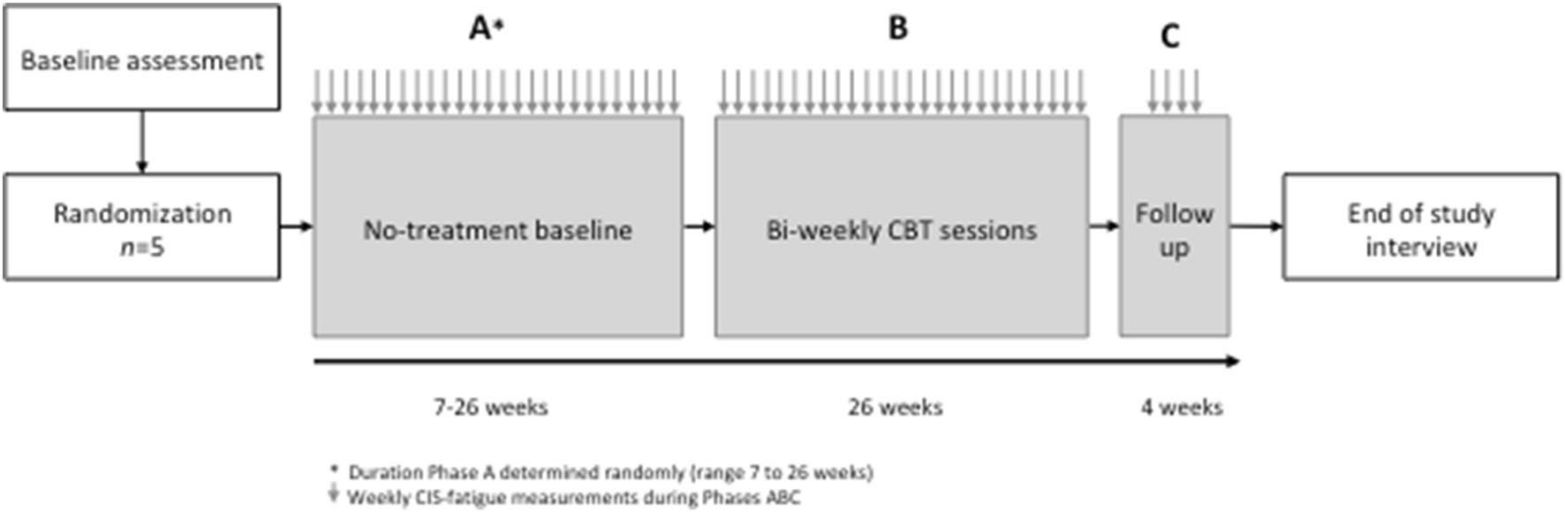
Study design

The study design is depicted in Fig. 1. Phase A represents the no-treatment baseline period with weekly measurements of fatigue. The duration of phase A was determined randomly with a computer-generated random number list and varied across participants (from 7 to 26 weeks). Allocation to baseline period duration was done by sealed envelopes, which were taken by an independent research assistant. Upon completion of phase A, participants received CBT for targeted therapy-related fatigue over a period of approximately 26 weeks (phase B). Participants continued to complete weekly measurements of fatigue during phase B. We administered four weekly follow-up measurements (phase C). Upon completion of phase C, an independent researcher who was not involved in the study (H.A.) conducted individual and semi-structured interviews exploring participants’ views on the effects of CBT for targeted therapy-related fatigue. All interviews were recorded and professionally transcribed.

### Measurements

At baseline, participants completed questionnaires on demographic characteristics and disease and treatment-related variables. We assessed fatigue severity with the fatigue severity subscale of the CIS (CIS-fatigue). The CIS-fatigue consists of 8 items scored on a 7-point Likert scale (range 8–56). The CIS-fatigue has been used in intervention studies testing the efficacy of CBT for post-cancer fatigue (Gielissen et al., 2006; Prinsen et al., 2013) and fatigue during adjuvant treatment (Goedendorp et al., 2010), and proved to be sensitive to change. The CIS-fatigue can differentiate between fatigue within normal limits and a clinically relevant level of fatigue. A cut-off score of 35 or higher is an indication for severe fatigue (Vercoulen et al., 1994; Worm-Smeitink et al., 2017).

### Intervention

Two clinical psychologists trained and experienced in CBT for cancer-related fatigue delivered the intervention. CBT for targeted therapy-related fatigue starts with psychoeducation about the cognitive behavioral model of cancer-related fatigue and formulation of treatment goals. The intervention aims to reduce severe fatigue and fatigue-related disability. Participants formulate goals in behavioral terms, such as resumption of work or recreational activities. Throughout the intervention, participants work toward attainment of the formulated goals. The decision to terminate the intervention is guided by achievement of formulated treatment goals. CBT for targeted therapy-related fatigue encompasses six intervention modules aimed at perpetuating factors of fatigue: (1) dysfunctional cognitions regarding CML and its treatment, including adherence to TKIs. Insufficient coping is targeted by talking or writing about these experiences (exposure) to help patients process the experiences and improve coping skills; (2) dysfunctional cognitions regarding fatigue. These cognitions, including catastrophizing, low self-efficacy, or unhelpful attributions, are discussed and more helpful ways of thinking are taught; (3) dysregulation of sleep–wake cycle. Patients are encouraged to maintain a regular sleep–wake pattern for a week with fixed bed and wake-up times and no daytime napping. If needed, additional sleep hygiene practices are discussed; (4) dysregulation of activities. We distinguished patients with fluctuating patterns of activity (i.e. bursts of activities followed by inactivity) and patients with a pattern of persistent inactivity. Patients with fluctuating patterns first establish a base level by evenly distributing their level of activity over the day. Upon reaching this base level, patients started a graded activity program (e.g. walking or cycling). Patients with persistent inactivity started the graded activity program immediately; (5) perceived lack of social support and unhelpful social expectations and responses of others. Some patients perceive a discrepancy between actual and desired social support, experience negative social interactions, or have unrealistic expectations of others. These patients are helped to instill more realistic expectations toward their social support group and to communicate more assertively with others; and (6) fear of increased disease activity. Some patients experience excessive fear of increased disease activity. Their fears and thoughts are discussed with a focus on how to deal with the uncertainty about their future health and response to TKI therapy. Dysfunctional beliefs are challenged and it is discussed how to reduce ruminating about the possibility of increased disease activity. Two weeks prior to the start of the intervention, participants completed questionnaires about their perpetuating factors and only the relevant CBT modules were included, thus creating a tailored intervention. CBT for fatigue is comprised of an average of 12.5 1-h sessions (Gielissen et al., 2006) and the number of sessions may vary among patients based on which modules are indicated. Sessions are scheduled more frequently in the beginning (e.g. weekly or bi-weekly) and the intervention slowly progresses to a lower intensity (e.g. every 3–4 weeks) when patients start integrating more elements into their everyday lives.

### Statistical analyses

Weekly measurements of fatigue were plotted for graphical inspection in terms of level, variability, and trend of data patterns. Descriptive statistics were used to calculate phase AB means for fatigue. To explore efficacy of the intervention, randomization tests for replicated AB single-case experimental designs were carried out (Edgington & Onghena, 2007; Onghena & Edgington, 2005). These nonparametric statistical analyses can be applied to single-case designs that have a random-assignment component. We used the difference between phase AB means as our test statistic and hypothesized that CBT would reduce fatigue severity. Visual and statistical analyses were performed using the R package for Single-Case Randomization Tests software [SCRT, version 3.0: University of Leuven, Belgium] (Bulte & Onghena, 2008). A one-tailed *p* value of less than .05 was considered statistically significant. Two study authors independently coded the interviews (H.P. and H.A.) and conducted thematic searches. Our thematic search focused on two specific topics of the text database: effects of CBT on severe fatigue and fatigue-related disability, and feasibility and acceptability of CBT.

## Results

Five patients with chronic-phase CML were enrolled in the study. One participant was excluded from the analysis because this person left the country and did not start the intervention after phase A. All four participants were Caucasian and three of the four participants were male (75%). Median age was 49 years (range 36–60) and median time since diagnosis was 3.5 years (range 0.8–15.4). Three participants were currently on Imatinib (400 mg once a day) and one on Bosutinib (600 mg once a day). No changes in CML treatment regimens occurred during the study period. However, two of the participants (3 and 4) were hospitalized during the end of the intervention for medical issues unrelated to their CML or its treatment. In addition, one participant (2) reported other psychosocial issues early during the intervention that were not identified during recruitment. To preserve anonymity, details regarding these issues are not further disclosed.

### Quantitative Results: Efficacy of CBT for Targeted Therapy-Related Fatigue

**Fig. 2:**
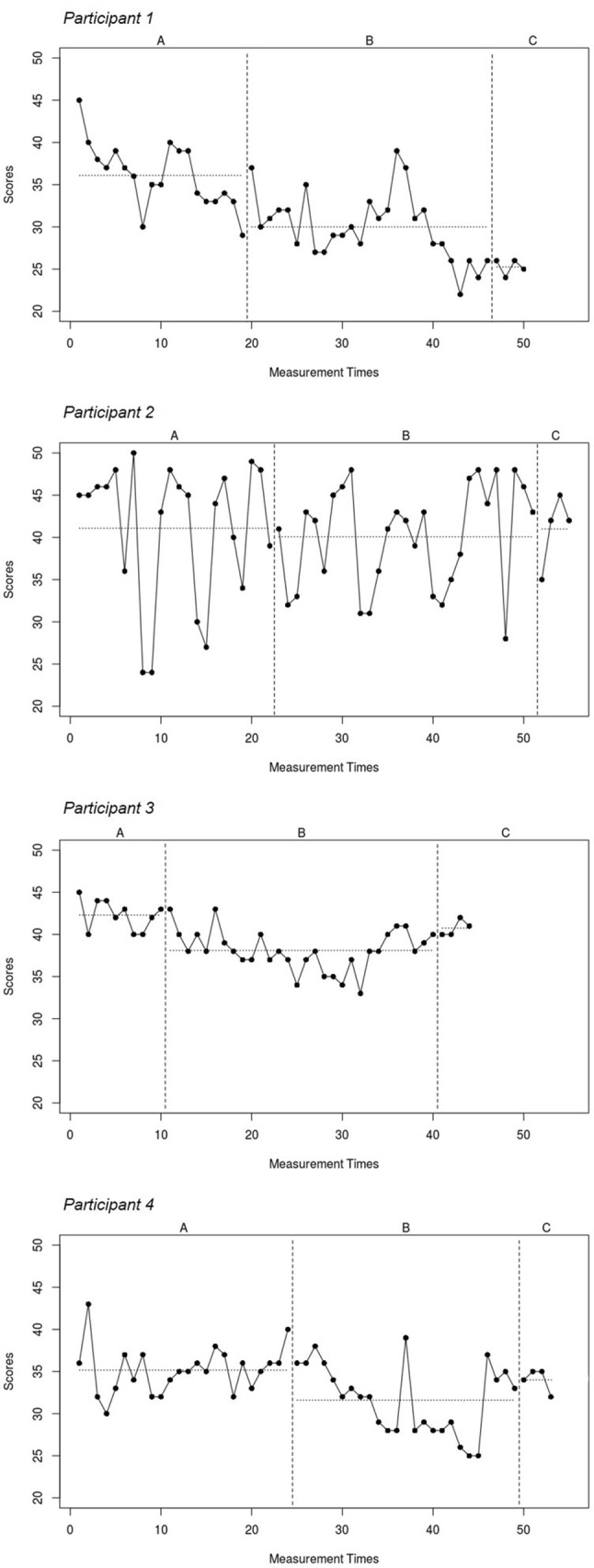
Weekly CIS fatigue scores within and across phases

**Fig. 3:**
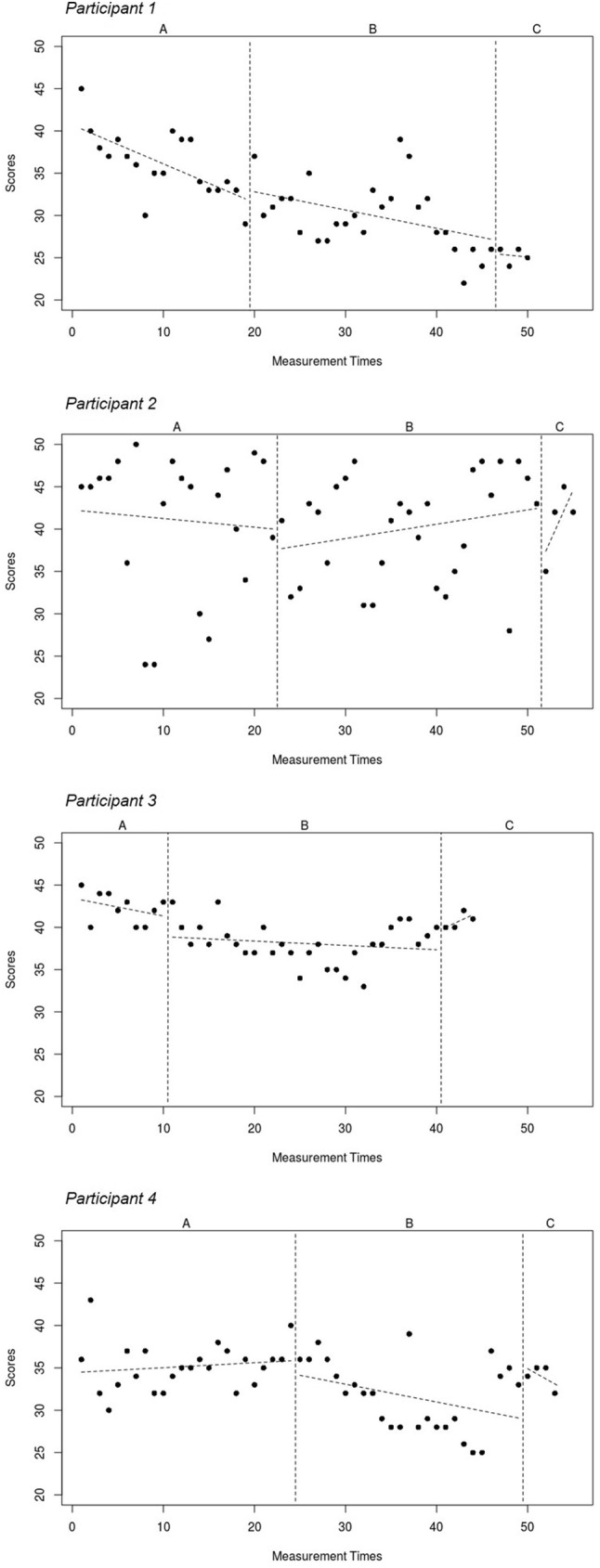
Least squares trend lines of CIS fatigue scores across time

Descriptive statistics of weekly CIS-fatigue scores are presented in Table 1. Phase A (no-treatment baseline) ranged between 10 and 24 weeks. CBT was provided over a period of 29–34 weeks and consisted of an average of 9.5 1-h sessions (range 6–13). Participants completed a total of 44 to 55 weekly fatigue measurements in phase A and B. Figures 2 and 3 show weekly CIS-fatigue scores and least squares trend lines across all measurement times (phases ABC). Phase A (no-treatment baseline) means were above the threshold for severe fatigue for all four participants. Visual inspection of line graphs, phase means, and least square trend lines for individual SCEs indicated downward trends in the expected direction for fatigue in phase B for two of the four participants (1 and 4). Notably, graphs of two participants (1 and 3) already indicated a slight downward trend of fatigue scores in phase A. The test statistics showed a decrease in the expected direction for each individual participant but the results from the randomization tests did not reach statistical significance (overall mean diff. phase AB = − 3.69, overall *p* = 0.18).

**Table 1:** Joint display of quantitative fatigue severity data and themes identified from qualitative data along with illustrative quotations

Subject	Mean diff. AB (SD)	No. of CBT sessions	Themes	Illustrative quotations
1	− 6.72 (5.14)	13	Improved coping with fatigue Reduced fatigue-related disability Improved quality of life	Now that I have more control over my energy levels, I also feel more confident to take on different tasks and activities I have been able to improve my daily routine. Before, I would stay in bed every day until 10am, and I thought I needed it. I am now able to get up at 7:30am and go to bed at 10:30 pm. During the hours in between, I have become much more productive. It is great to be able to experience the entire morning instead of starting my day around lunch time My efficiency is higher and, with that, my quality of life is also better. I know much better what I can and can’t do. I feel a bit more comfortable in my own skin
2	− 0.91 (6.89)	7	Improved coping with fatigue Reduced fatigue-related disability	The most important thing for me, that will have the biggest impact on me, is, I think, just the acceptance It was really helpful to create a daily routine and put structure into my life
3	− 3.89 (2.83)	11	Improved coping with fatigue Reduced fatigue-related disability Improved quality of life	The fatigue is still present. But you can learn how to cope with it differently. I have learned to cope with it much better. (..) When I look at myself in the mirror, I am thinking: I don’t see a man who is always preoccupied with being fatigued anymore. I have the space to be busy with other things Before I started this intervention, I went to bed every afternoon to rest. After work, I went to bed to rest. Now, I never go to bed in the afternoon anymore. I just live from 7am until 11 pm. So, I have more time during the day to undertake several activities I have learned to give more and enjoy the things in life more, the things that are most important to me. And because of that, it also improves quality of life, I think
4	− 3.24 (3.83)	6	Improved coping with fatigue Reduced fatigue-related disability Improved quality of life	I am better able to cope with the fatigue. Well, not at present because of my [unrelated medical issue], but before I could do much more during the day and that for several days in a row. Before the intervention I could push through for three days and then I would crash for a day I am better at dosing out my activities. I think I can do more because I no longer do all my activities one after the other The fatigue is an issue. It can make life more pleasant when you suffer less from fatigue. (..) The opportunities I have, the things I can do, those have increased. As in I actually can do that, I am living more. I have more life

### Qualitative Results: Effects of CBT on Fatigue and Other Domains

All four participants completed individual interviews after phase C. Table 1 provides a joint display of quantitative and qualitative data for each participant. One participant (4) reported a substantial transitory reduction in severe fatigue. The other three participants (1–3) did not explicitly mention a substantial reduction in fatigue severity. However, all four participants reported that they felt they were better able to cope with the fatigue (see illustrative quotations in Table 1). Two participants (2 and 3) reported that the intervention had helped them to accept the fatigue. All four participants found that after the intervention they optimized their daily structure and accomplished more during the day. Finally, three participants (1, 3, and 4) experienced improved quality of life because of changes brought on by the intervention, such as feeling less tired and experiencing less fatigue interference.

### Feasibility and Acceptability of CBT for Targeted Therapy-Related Fatigue

Feedback from participants indicated that the intervention was feasible and acceptable for use in this patient population. When participants were asked how they would rate their level of satisfaction with the intervention on a scale of 0–10; all patients gave a satisfaction rate of 8 and would recommend it to other severely fatigued CML patients.

## Discussion

We used a mixed-method convergent design to evaluate the feasibility and efficacy of a fatigue-specific CBT intervention through SCEs and semi-structured interviews. The objective data showed a decrease in the expected direction for fatigue severity in all four participants but the overall findings did not indicate a significant intervention effect. Participants did report qualitative improvements; they were better able to cope with fatigue, experienced less interference by the fatigue with daily functioning, and most reported improved quality of life. The intervention was acceptable for patients and feasible.

Previous studies with CBT in disease-free cancer survivors showed that most patients were no longer severely fatigued following treatment (Abrahams et al., 2017; Gielissen et al., 2006; Prinsen et al., 2013). The findings of the present study are different, with a decrease in fatigue in the expected direction but no significant reduction. Our study participants reported highly variable fatigue ratings across weeks. Intervention studies on cancer-related fatigue typically include a pre/post-treatment assessment to determine treatment responsiveness and do not provide more detailed assessments of fatigue over time. More research into the variability of fatigue would be helpful to further our understanding of this complex and multifactorial symptom. In addition, two of the four participants were hospitalized toward the end of the intervention because of medical events that were unrelated to CML and its treatment. It is possible that mean fatigue scores during phase B were significantly affected by these events and interviews confirmed that both patients experienced a setback because of these hospitalizations. The impact of these events on their fatigue scores may have limited our ability to demonstrate objective intervention effects. Alternatively, variability and trends in the direction of the expected effect during phase A may have prevented us from establishing a representative baseline, which is important in SCE research because it is used to be compared with phase B (Smith, 2012). Furthermore, the observed pre-intervention period trends in two patients could reflect anticipation effects.

Fatigue is known to be one of the most common side effects of TKIs (Efficace et al., 2013; Phillips et al., 2013; Williams et al., 2013). Patients reported strong somatic attributions in relation to the cause of their fatigue, i.e. contributing it to the use of TKIs primarily. According to the cognitive behavioral model of post-cancer fatigue (Gielissen et al., 2006; Servaes et al., 2007), cancer and its treatment are triggers for fatigue but after completion of treatment the perpetuating factors are responsible for the persistence of severe fatigue. However, in CML patients on chronic TKI treatment, the trigger for fatigue remains present and, therefore, it may be unrealistic to expect to reduce fatigue to normal levels. Yet, all patients reported qualitative improvements in coping with fatigue and fatigue-related disability. In addition, for three patients, quality of life improved. Despite the persistent severity of fatigue, improved coping, level of functioning, and quality of life are still high-priority goals. When CML patients continue to receive TKI treatment for years and potentially the rest of their lives, the preservation of physical and emotional well-being is paramount. In addition, other patient populations on chronic cancer treatment with targeted therapies may also benefit from this adapted intervention. While we should be cautious in generalizing the current results to other populations, it is worthwhile to test the efficacy of this intervention in the increasing number of patients whose cancer is kept in check with new targeted therapies, e.g. patients on PARP inhibitors for ovarian cancer.

To our knowledge, this is the first study designed to test the effects of an intervention aimed specifically at reducing targeted therapy-related fatigue, the most important factor impacting quality of life in patients with CML. Qualitative feedback from participants indicated that the intervention was perceived as useful and acceptable for use in this patient population. Patient satisfaction with the received intervention was high and participants would recommend it to other severely fatigued CML patients. The use of a mixed methods design is a major strength of the current study, as the integration of the quantitative and qualitative data resulted in a broader understanding of the effects of CBT on targeted therapy-related fatigue.

## Limitations

While the SCE approach is particularly appropriate when unaddressed areas are explored and small-*n* populations such as patients with CML are studied, our study has some limitations. First, we had a smaller sample size than anticipated (i.e. four completed SCEs instead of five), which may have resulted in limited power to demonstrate significant overall intervention effects. Second, participants in our study had a median age that was 15 years younger than the average age of patients with CML (i.e. 64 years). Third, the SCE design is less robust to deal with the impact of unrelated medical or psychosocial events on fatigue scores compared to a randomized controlled trial (RCT). Fourth, we did not assess intervention fidelity or participant engagement with the different intervention modules in this initial small-scale study. Lastly, we did not include weekly measurements of outcomes other than fatigue; a decision that had been made carefully to minimize the burden of participation. However, it is possible that effects on emotional and physical functioning may have been seen when measures were included, as found in previous fatigue intervention trials for other cancer populations (Gielissen et al., 2006; Goedendorp et al., 2010). In light of this, the mixed methods design was particularly valuable and enabled us to capture these important qualitative findings.

## Conclusions

In sum, our study provided preliminary evidence for the feasibility and acceptability of CBT for severely fatigued CML patients receiving targeted therapy. Clinical psychologists could integrate knowledge on perpetuating factors for fatigue to educate patients on oral treatment for cancer who experience severe fatigue. Although we could not demonstrate an overall objective intervention effect in this small-scale study, qualitative findings indicated that the intervention is feasible and shows promise for improving emotional and physical functioning of CML patients. Further large-scale research (i.e. an RCT) is needed to determine efficacy of the intervention to reduce fatigue severity and improve adherence to TKIs, as well as to explore optimal fatigue intervention delivery in the population of CML patients receiving TKI treatment. A pilot RCT is currently underway to determine the effects of our adapted intervention in a larger sample.
